# On-Chip Curing by Microwave for Long Term Usage of Electronic Devices in Harsh Environments

**DOI:** 10.1038/s41598-018-33309-x

**Published:** 2018-10-08

**Authors:** Jun-Young Park, Weon-Guk Kim, Hagyoul Bae, Ik Kyeong Jin, Da-Jin Kim, Hwon Im, Il-Woong Tcho, Yang-Kyu Choi

**Affiliations:** School of Electrical Engineering, Korea Advanced Institute of Science and Technology, (KAIST) 291 Daehak-ro, Yuseong-gu, Daejeon, 34141 Republic of Korea

## Abstract

Microwave-induced thermal curing is demonstrated to improve the reliability and to prolong the lifetime of chips containing nanoscale electron devices. A film containing graphite powder with high microwave absorbing efficiency was fabricated at low cost. The film is flexible, bendable, foldable, and attachable to a chip. A commercial off-the-shelf chip and a representative 3-dimensional (3D) metal-oxide-semiconductor field-effect transistor (MOSFET), known as FinFET, were utilized to verify the curing behaviors of the microwave-induced heat treatment. The heat effectively cured not only total ionizing dose (TID) damage from the external environment, but also internal electrical stress such as hot-carrier injection (HCI), which are representative sources of damages in MOSFET insulators. Then, the characteristics of the pre- and post-curing electron devices are investigated using electrical measurements and numerical simulations.

## Introduction

Improving the reliability and stability of chips containing more than a billion electronic devices is one of the challenging issues that affect their long-term operation. While electron devices like the bipolar junction transistor (BJT) are solely composed of silicon and have no gate insulator, the inherent operating principle of the metal-oxide-semiconductor field-effect transistor (MOSFET) requires the use of a gate insulator. The MOSFET is considered as an element of logic circuits because it combines low power operation and scalability with high speed and excellent gain.

Since the MOSFET is based on the use of a gate electrode, device degradation due to damage to the gate insulator is unavoidable. Unwanted high energy irradiation, such as x-rays or γ-rays from external environments, is particularly detrimental to device performance and reliability. For example, exposure to a total-ionizing dose (TID) will ionize various insulators used for electrical isolation in the MOSFET, and degrade the dielectric quality^1^. When the device-to-device isolation layer formed by the local oxidation of silicon (LOCOS) or shallow-trenched isolation (STI) is damaged by TID, the off-state leakage current (*I*_OFF_) of the MOSFET is increased due to the accumulated isolated parasitic positive charges^2^. Moreover, as the parasitic charges accumulate at the gate insulator or the gate spacer, the threshold voltage (*V*_TH_) of the MOSFET is shifted, and as a consequence, logic circuits can suffer from unexpected malfunctions^2,3^.

In addition to the abovementioned external causes of device degradation, internal stresses produced by hot-carrier injection (HCI) or Fowler–Nordheim (FN) tunneling can also accelerate device aging, and lead to device failure. Typically, damage of the gate insulator by HCI and FN tunneling increases *V*_TH_ as well as sub-threshold swing (*SS*), and decreases on-state drain current (*I*_ON_) in a n-channel MOSFET. Increasing *V*_TH_ can lead to a *V*_TH_ mismatch in the logic circuits and reduce the refresh time of dynamic random access memory (DRAM)^4^. In addition, increasing *SS* can increase the *I*_OFF_ as well^5^. And as MOSFETs continue to be scaled down for better performance and higher packing density, the abovementioned degradation and aging issues by external or internal causes become ever more serious. Because of the increase in the lateral electric field in a short-channel MOSFET, more hot-carriers are generated compared to a long-channel MOSFET. Moreover, as the gate length (*L*_G_) of the MOSFET is reduced, the TID also becomes much more severe^6^.

Recently, a novel method to restore device performance and reliability after damage was suggested. We previously reported a self-curing MOSFET which could cure TID, HCI, and FN stress-induced device damage^3,5,7^. To cure the damaged insulators, localized high temperature was generated by an embedded nanowire-heater in the MOSFET. In operation, the generated heat was transferred to the TID damaged insulators, electrons in the insulators were thermally excited, and then neutralized by the parasitic positive charges^1,5^. In addition, the heat accelerated the diffusion of residual hydrogen ions, which inherently existed in the device, thus curing damage to the insulators caused by HCI and FN stress^8^. In this way, the degraded device performance and reliability were successfully recovered.

However, the reported curable MOSFET has a fundamental limitation, because the method demanded the fabrication of additional electrodes to generate Joule heat. This unavoidably lowered layout efficiency. Moreover, because it required an additional electrical signal for device curing, controlling the configuration of the input signal (voltage) could be quite complicated.

In contrast to the abovementioned recovery method in a unit transistor level, other groups suggested recovery methods utilizing an embedded heater module, which was intercalated into a package^9–13^, i.e., interior heater. In terms of layout efficiency, the recovery method in the package level is more preferred than that in the transistor level. But, the former still accompanies with extra fabrication of heater module including temperature sensor and its assembly and is not a cost-effective approach, either. In addition, it demanded relatively high power for heating and the induced heat distribution was not uniform^9^. Hence longer recovery time was required. Alternatively, the recovery method in the package level with an external heater, i.e., exterior heater was explored by use of a hot-chuck underneath a chip^14^, however, its practicality was still modest. Comparison of the various recovery methods is as summarized in Table S1 (Supplementary Information). It is timely to develop another recovery method in the chip level with low-cost and fast curing without extra design modification.

This work proposes an alternative and evolved method, microwave-induced on-chip curing, which is not transistor level curing, to ensure the long term usability of the chip. Microwave heating is one of the most promising and sustainable techniques for the annealing and synthesis of materials because it features fast heating, high product yield, applies uniform temperature, and can be performed at atmospheric pressure^15–21^. The proposed method does not require an additionally fabricated heater which would introduce high complexity and low layout efficiency.

A microwave absorbable film and a commercial microwave generator were employed to prolong the lifetime of the chip, even in harsh environments. The film is composed of a high efficiency microwave absorption material such as graphite. The heat induced by the microwave irradiation cures the damage in electron devices produced during operation by external and internal causes. Typically, external causes such as γ-ray radiation is much more widespread and severer, the recovery from the γ-ray radiation is much difficult than that from the HCI and FN stress.

This work can contribute to improving the performance and reliability of a chip which has been exposed to external penetrating energy, or has been frequently exposed to internal electrical stresses beyond a transistor. At the same time, the proposed method allows malfunctioning devices to be reused, and the lifespan of the chip can be prolonged without need of replacement.

## Results and Discussion

**Figure 1:**
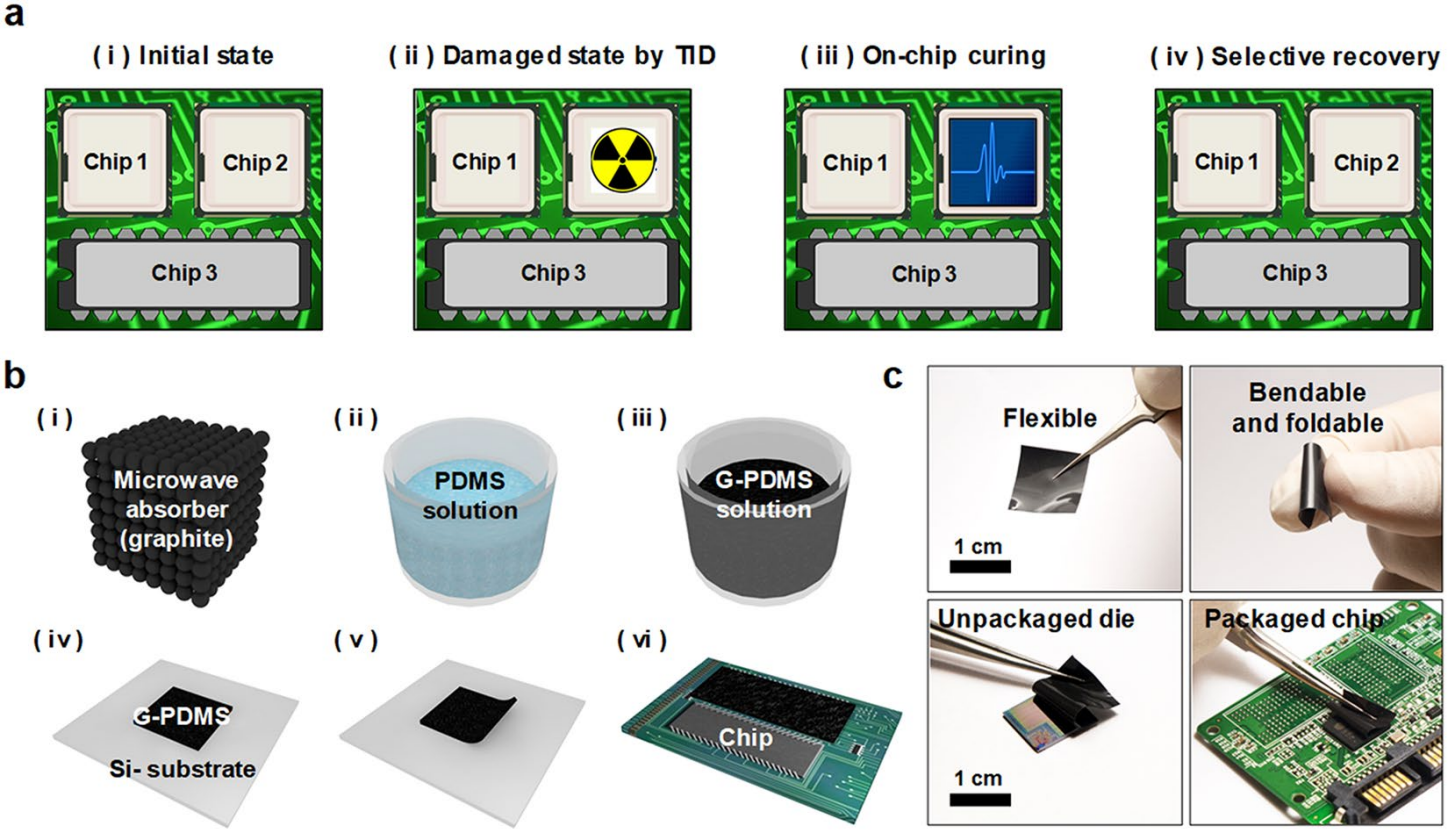
(**a**) Operating principle of the proposed on-chip based microwave curing (MwC). (**b**) Fabrication flow of the microwave absorable film. (i) graphite powder was selected as a microwave absorber, (ii) 10:1 PDMS solution was used as the elastic binder material, (iii) 10% of the weight ratio of graphite powder was added to the mixed PDMS solution, and poured onto the silicon substrate, (iv) spin-coating was used to make a film, (v) curing in an oven, and detaching the G-PDMS (vi) cutting the film to be fitted to the chip size and attached to a damaged chip, (**c**) optical photograph of the fabricated G-PDMS film.

Figure 1(a) shows the operating principle of the proposed method for repairing electron devices using microwave curing (MwC). (i) On a board such as a motherboard, various kinds of electron devices (packaged chips) including CMOS are assembled for each function. (ii) However, as the operating time of the chip increases, the electron devices are stressed by energy coming from the external environment. Some vulnerable components of the chip (chip 2 in Fig. 1(a)) may be affected by the radiation energy and gradually damaged. At the same time, the frequent application of electrical stresses can also degrade the chip, even when the devices are operated under normal conditions. Such damages can become serious enough to shorten the stable operating lifetime of the chip. (iii) In order to cure such damages, a microwave absorbable film was attached to the surface of the damaged chip, and the entire board was irradiated with microwaves generated by a commercial microwave generator. Because of the relative difference in microwave absorbing capacity of the materials, the irradiated microwaves are selectively absorbed on the targeted chip. Through the selective chip recovery, it is preferred to cure a damaged chip solely rather than to replace all the chips on the board. Additionally, a price of a board capable of being endurable against radiation is much more expensive than that of a conventional board. Hence selective annealing can contribute to reducing the cost for longtime usage. (iv) As the microwave energy is absorbed by the film, a low temperature of less than 250 °C is produced on the exterior surface of the chip, and the heat cures the degraded chip without decomposition of outer packaging such as epoxy, interconnection, soldering, and printed circuit board (PCB) (Supplementary Information Fig. S1). The curing characteristics are proportional to the temperature and time, and the conditions can be controlled^5^.

Figure 1(b) shows a schematic of the fabrication process of the abovementioned microwave absorbable film. Carbon based materials such as carbon nanotube (CNT), activated carbon, and graphite have high microwave absorbing characteristics due to their dipolar polarization^21–23^. We selected and used graphite powder as an absorbing material, because graphite is commercially available and lower priced than the others. To fabricate the graphite-PDMS (G-DPMS) film, a base solution of polydimethylsiloxane (PDMS, model: Sylgard 184, Dow corning) and its curing solution were mixed at a weight ratio of 10:1. Then, 10% weight ratio of graphite powder (Sigma-Aldrich Chemistry) was added to the mixed PDMS solution and stired until the powder was sufficiently mixed. Afterwards, the liquid mixture was poured onto a dummy silicon wafer, spin-coated at 2000 rpm for 2 sec, and baked at 85 °C for 30 min. If the graphite powder is used alone without the use of a sol- or gel-phase material such as PDMS, the powder is dusty, and floats in air, thus is difficult to handle. All of the processes for fabricating the microwave absorbing film were carried out in a low-cost facility, e.g., without a vacuum process. The fabricated G-PDMS film is flexible, bendable, foldable, and easily attached to the chip, as shown in Fig. 1(c). The microwave absorbing capability of the G-PDMS can be controlled by changing the weight ratio of graphite or the PDMS curing solution.

**Figure 2:**
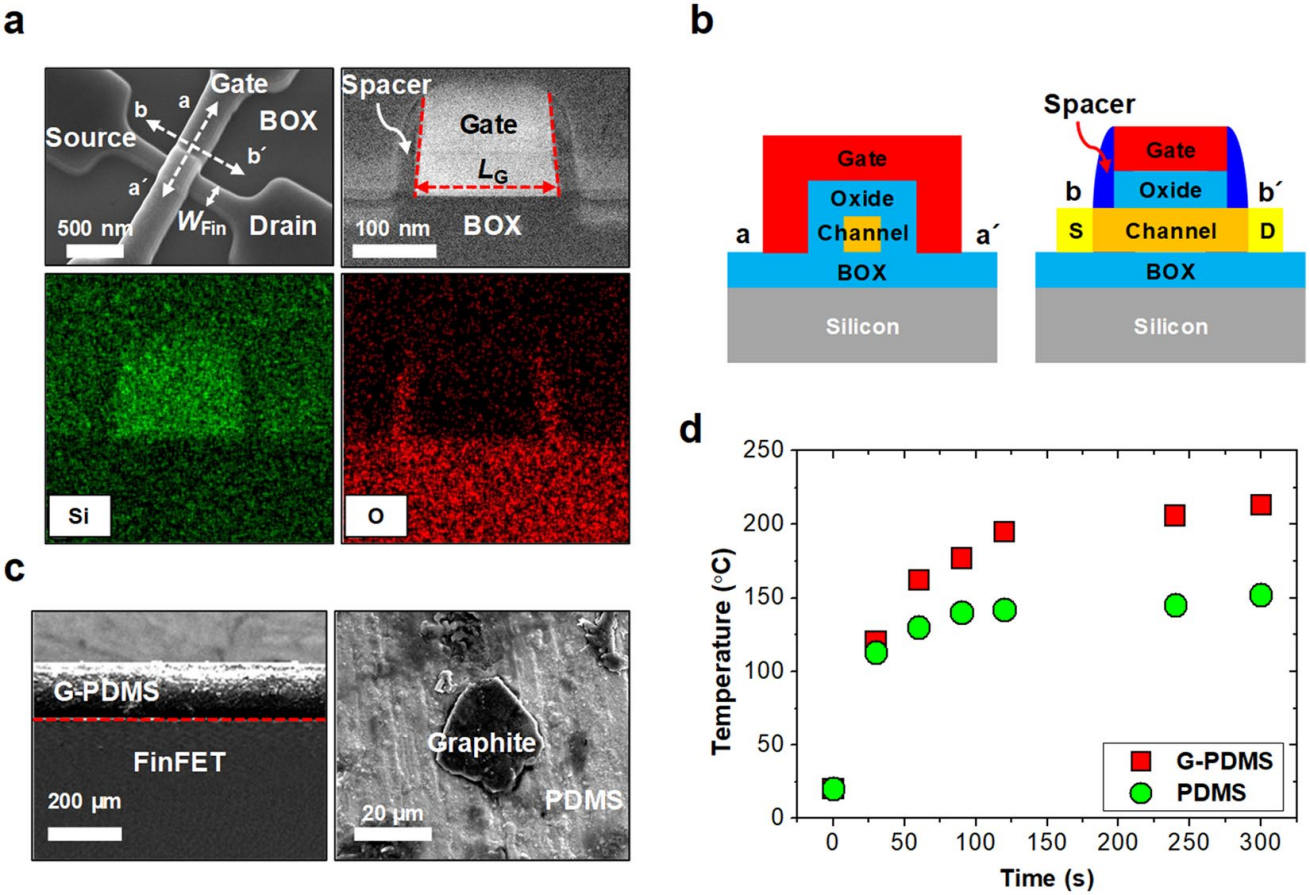
(**a**) Cross-sectional TEM images and energy-dispersive spectrometer (EDS) mapping of the FinFET along the b-b′ direction. (**b**) Schematic of the FinFET to observe the curing behavior of on-chip based MwC. (**c**) Cross-sectional and top-view SEM image of the fabricated G-PDMS film attached to the unpackaged FinFET die. The graphite particle size was less than 20 µm. (**d**) Measured surface temperature of the G-PDMS on the unpackaged die, after microwave irradiation.

Figure 2(a) shows scanning-electron microscope (SEM) and transmission-electron microscope (TEM) images of the fabricated platform device, known as a FinFET, which is a representative 3-dimensional (3D) MOSFET used as a unit element for logic or memory function. The nominal channel width (*W*_Fin_) of the FinFET is 160 nm, its gate length (*L*_G_) is 180 nm, and its gate spacer width (*W*_Spacer_) is 25 nm. Figure 2(b) shows the schematics of the FinFET. The device was fabricated on a silicon-on-insulator (SOI) substrate, and all the processes are completely compatible with the standard complementary metal–oxide–semiconductor (CMOS) processes. Detailed descriptions of the fabrication process flow are summarized in the Supplementary Information Fig. S2.

Figure 2(c) shows a cross-sectional SEM image of the G-PDMS film, which is attached on an unpackaged die containing numerous FinFETs. The thickness of the G-PDMS film is approximately 180 µm and can be simply controlled by changing the rotation speed of the liquid-phase spin-coating process. The particle size of the graphite is approximately less than 20 µm. Figure 2(d) shows the measured temperature of two samples: PDMS and G-PDMS, which were described in Fig. 2(c), after irradiation with microwaves. A pure PDMS film without the graphite powder was also fabricated as a control. The frequency of the microwaves was 2.45 GHz, and the power was 800 W (model: MWO-2018, LG electronics). After the microwave irradiation, the surface temperature of the G-PDMS was measured using a non-contact infrared thermometer (model: DT8380, Tekpower). As expected, the G-PDMS showed a higher temperature than the pure PDMS, as shown in Fig. 2(d). If the weight ratio of the graphite powder is increased by adding chloroform during the mixing process^24^, the temperature increment is intensified.

**Figure 3:**
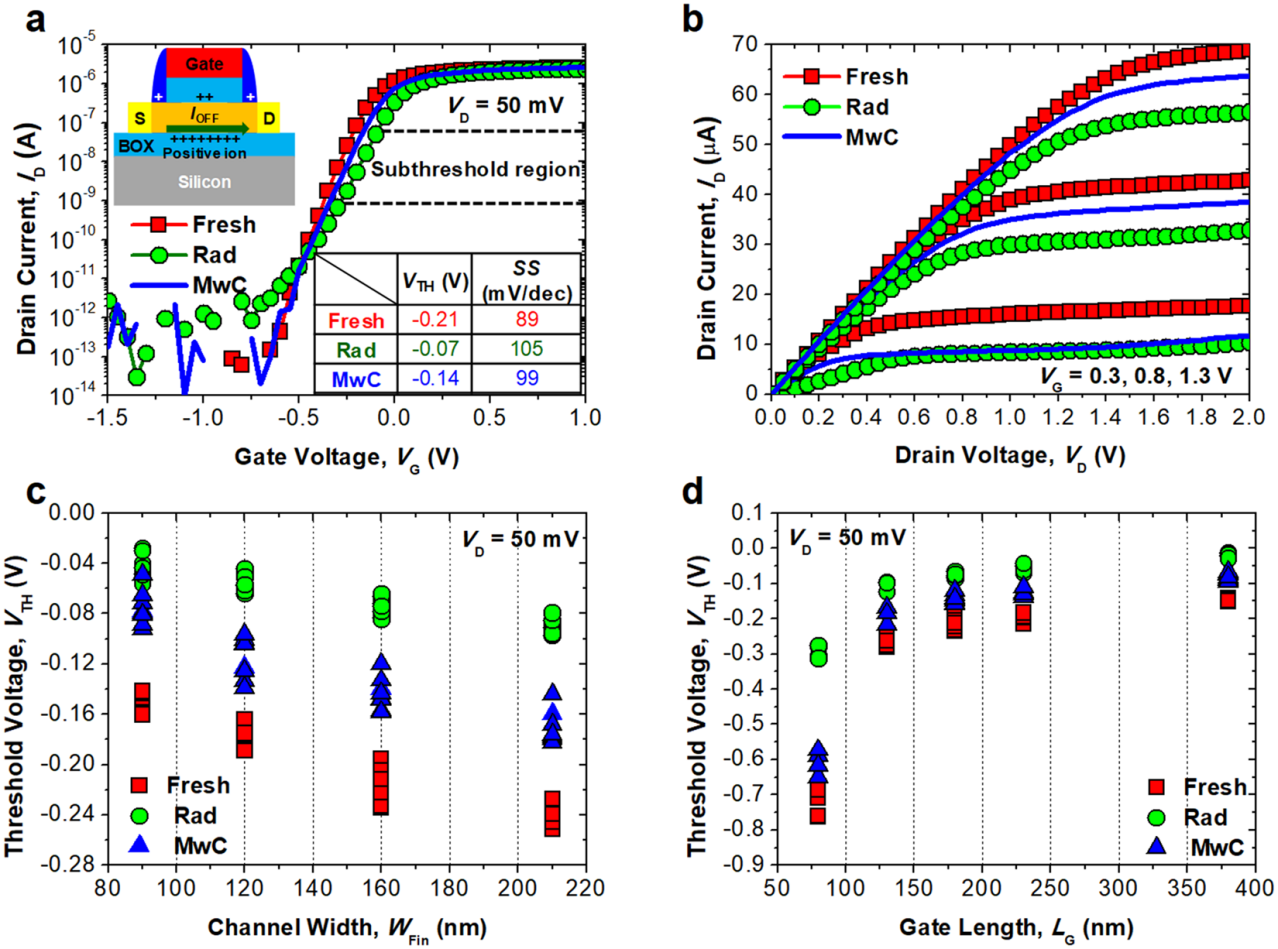
(**a**) Measured *I*_D_-*V*_G_ characteristics of the fabricated FinFET, at an initial state, after γ-ray exposure, and after microwave exposure for 60 sec. Inset image shows the location of insulator damage in the FinFET. Inset table shows the extracted *V*_TH_ and *SS* from the *I*_D_-*V*_G_. (**b**) Measured *I*_D_-*V*_D_ characteristics to observe the output current performance. (**c**,**d**) Extracted *V*_TH_ of the fabricated FinFET with various *W*_Fin_ and *L*_G_ at the initial state, before the MwC, and after the MwC.

Figure 3 shows the measured electrical characteristics of the fabricated FinFET after external damage by TID. Electrical measurements before and after applying intentional TID damage to the insulators of the FinFET were carried out with a semiconductor parameter analyzer (B1500A) under air ambient at room temperature. After irradiation by the microwave generator for 60 sec, the FinFETs were cured. The drain current (*I*_D_), which is the current flow from the drain to the source, was measured before and after the TID in order to compare the performance degradation, as shown in Fig. 3(a). Then, the threshold voltage (*V*_TH_), which is the criterion point of gate voltage (*V*_G_) used to determine the on-state and off-state of the transistor, was extracted^25^. Detailed explanations of the radiation experiment are described in the above experimental section. When high energy rays from an external radioactive source (^60^Co) interact with the insulators used to electrically isolate the FinFETs, they are ionized. During the ionization of the insulators, electrons and holes are generated, and the electrons are diffused and move out to the gate of the FinFET. However, the holes cannot be removed promptly because they have lower mobility in the insulator compared with electrons, and they remain trapped at the interface between the insulator and silicon. The trapped holes play a role in increasing parasitic positive charges during device operation. The inset of Fig. 3(a) shows the location where parasitic charges have accumulated.

Typically, the interfaces of the gate spacer, gate insulator, and buried oxide (BOX) in the SOI are damaged by the TID. In addition, the TID-induced damages are usually proportional to the thickness of the insulator, because not only the electrons but also holes can be easily removed in thin insulators^1,2^. Accordingly, in this case, the positive charges and damages generated by the TID were dominant in the BOX layer in the SOI wafer, because the thickness of the BOX (400 nm) is much thicker than that of the other insulator layers, such as the gate spacer (25 nm) and the gate insulator (5 nm).

Figure 3(a) shows the measured *I*_D_-*V*_G_ characteristics of the FinFET. After the TID, the sub-threshold swing (*SS*) of the device was degraded from 89 mV/dec to 105 mV/dec due to the damage to the BOX, and off-state leakage current was increased. However, when the MwC was carried out with the aid of a G-PDMS film, the degraded electrical performance was recovered. Figure 3(b) shows the output characteristics of the FinFET after the MwC. Due to the curing of the *SS*, the output performance of *I*_D_, was also recovered and returned close to its initial state.

Figure 3(c,d) show the extracted *V*_TH_ of the FinFET before and after the MwC. It should be noted that the *V*_TH_ is one of the most important metrics used in radiation hardening^26^. Various sizes (8 samples) of FinFET were characterized. After the TID, the *V*_TH_ was shifted rightward due to the degraded *SS*. Such degradation becomes more severe as the *W*_Fin_ becomes wider and the *L*_G_ becomes shorter. These degradations arise from the effects of the TID and are worsened by the short-channel effects of the FinFET. This observed trend is consistent with previously reported work^6^. After application of the TID, the G-PDMS film was attached to the unpackaged FinFET embedded die, then microwave irradiation for on-chip curing was performed for 60 sec. Thereafter, the G-PDMS film was removed, and the *I*_D_ was re-measured to observe the post-curing behaviors of the FinFET. When the G-PDMS is detached, grounding the die is helpful to avoid unwanted electro-static induced damage to the gate insulator, as shown in Supplementary Information Fig. S3. After the MwC, the degraded *V*_TH_ was recovered in the variously sized FinFETs, thanks to the curing of damages in the BOX. The proposed method can be utilized regardless of the transistor geometry or size.

**Figure 4:**
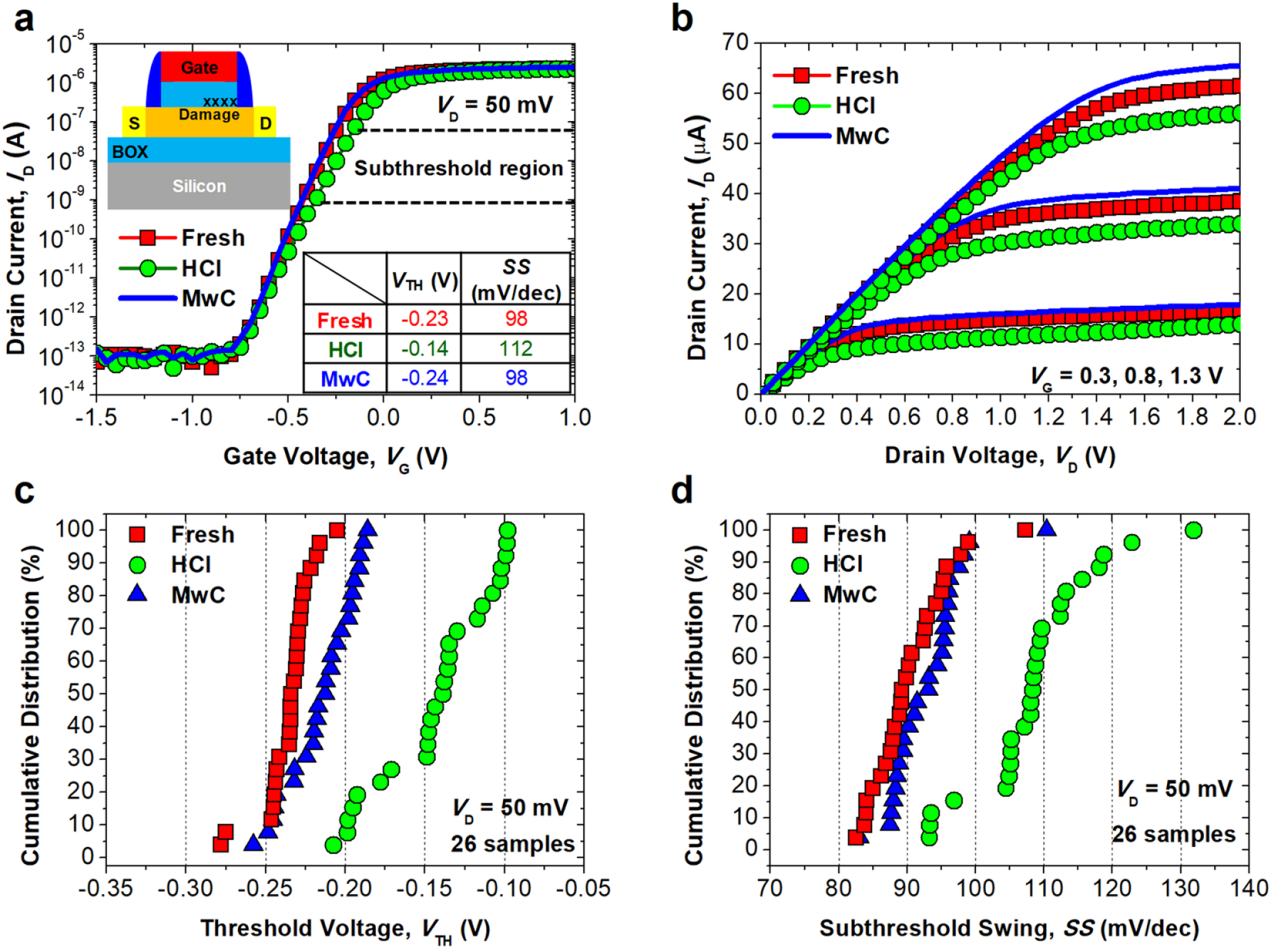
(**a**) Measured *I*_D_-*V*_G_ characteristics of the fabricated FinFET at the initial state, after the HCI, and after microwave exposure for 60 sec. Inset image shows the location of insulator damage in the FinFET. Inset table shows the extracted metrics *V*_TH_ and *SS* from the *I*_D_-*V*_G_. (**b**) Measured *I*_D_-*V*_D_ characteristics to observe the output current performance. (**c**,**d**) Cumulative distribution of extracted *V*_TH_ and *SS* of the FinFET at the initial state, before MwC, and after MwC.

Figure 4 shows the measured electrical characteristics of the FinFET, to observe the effect of the curing behavior of the proposed method on the internal stress stemming from device operation. When a chip is operated for a long period of time, some of the electrons which flow in the channel of the unit transistor become trapped at the gate insulator, or break the interface between the channel and the gate insulator^27,28^, as shown in the inset of Fig. 4(a). To mimic this electrical stress condition, hot-carrier injection (HCI) was intentionally applied to the device to accelerate device aging. The source voltage (*V*_S_) of ground, drain voltage (*V*_D_) of 6 V, and gate voltage (*V*_G_) of 3 V were applied for 20 sec. After the HCI, the abovementioned procedures described in the above paragraph were repeated for MwC. Figure 4a shows the measured *I*_D_ with various *V*_G_. Due to the increased *V*_TH_, the *I*_D_ was reduced after the HCI. However, after the MwC, the degraded output performance (*I*_D_), was recovered. Sometimes, the recovered *I*_D_ is better than the pristine *I*_D_. It can be inferred that pre-existing damages in the gate insulator caused by the fabrication processes are also cured by the proposed MwC. And the degraded *V*_TH_ and *SS* were recovered with barely changed distribution, as shown in Fig. 4(c,d).

**Figure 5:**
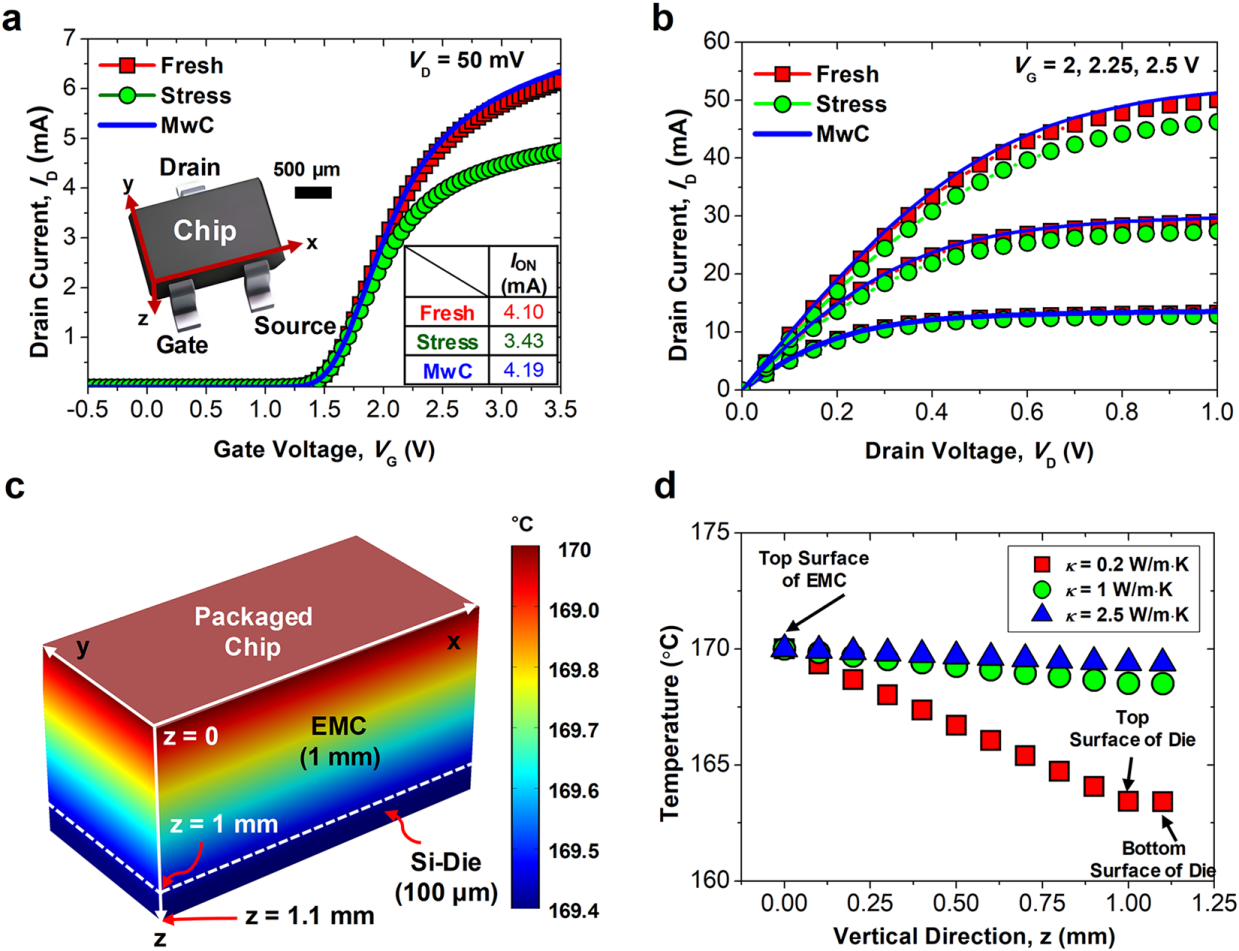
(**a**) Measured *I*_D_-*V*_G_ characteristics of the commercial off-the-shelf chip (model: SOT-323, ROHM Semiconductor) at the initial state, after the stress, and after microwave exposure for 60 sec. Inset shows an image of the chip. (**b**) Measured *I*_D_-*V*_D_ characteristics to observe the output current performance. (**c**) Simulated heat distribution profile of a packaged chip of (**a**) under microwave irradiation. (**d**) Extracted temperature of (**c**) along the vertical direction (z) of the chip with various *κ* of the EMC.

Figure 5(a,b) show the measured *I*_D_ of the commercial off-the-shelf chip (model: SOT-323, ROHM Semiconductor) used to verify the practicality of the proposed method. The size of the chip is 2 mm (length) × 1 mm (width) × 1 mm (height), and contains a packing layer of epoxy molding compounds (EMC). After measuring the pristine chip, the source voltage (*V*_S_) of ground, drain voltage (*V*_D_) of 50 mV, and gate voltage (*V*_G_) of 3.5 V were applied for 1200 sec to generate 15% performance degradation. After the stress, the G-PDMS film was attached to the surface of the chip, and irradiated with microwaves for 60 sec for on-chip curing. The *I*_D_ was re-measured. The *I*_ON_ of the chip was extracted at *V*_G_ = *V*_TH_ + 1 V for comparison of annealing behaviors. It can be seen that the proposed MwC is still effective, even though the die was covered with a 1 mm EMC layer. Moreover, the recovered *I*_ON_ of the chip was also better than the pristine *I*_ON_, and this effect is consistent with the results described in Fig. 4.

The chip is completely packaged by the EMC, which plays role in protecting it from the molecules in air. Hence it can be deduced that the recovery mechanism is governed not by exterior gas but by interior hydrogen. Figure 5(c) shows the simulated heat distribution profile of the chip shown in the inset image of Fig. 5(a). Detailed explanations and information about the simulation are described in the below methods section. Temperature of 170 °C was applied to the top surface of the EMC. Figure 5(d) shows the extracted temperature of the packaged chip along the vertical direction. Even though the thermal conductivity (*κ*) of the EMC was 0.2 W/m∙K, the transferred temperature was still higher than 160 °C.

This temperature was high enough to cure the electron devices (MOSFETs) located underneath the EMC. In addition, if the *κ* of the EMC is further increased by material engineering^29^, the heat transfer efficiency inside the package can also be further enhanced.

In this study, on-chip curing was demonstrated using a commercial microwave generator. To enhance the microwave absorbing efficiency, a film composed of graphite powder and flexible PDMS was fabricated and applied to the chip, to concentrate the heating effect on the damage. The film was flexible, bendable, foldable, and attachable, and its fabrication cost was low. The curing behaviors of the chip were verified by experiments and supportive simulations. This on-chip curing restored degraded device performance, such as *I*_D_, *SS*, *V*_TH_ to nearly their pristine state. This curing effect was verified using both an unpackaged die and a packaged chip. The proposed method makes it possible to prolong the lifespan of electron devices subjected to external and internal stresses. This concept can be used for not only for a high-level commercial electronic device working in harsh environments, and but also for a low-level personal electronic device which requires long-term reliability. However, there may be possibility that the proposed recovery method can accelerate device variability, because the MwC can over-anneal a normal device, which does not require the recovery. Hence optimization of the annealing condition may be needed to minimize such side effect.

## Methods

### Thermal simulation of a chip

After the device fabrication, a semiconductor parameter analyzer (B1500A) was used at room temperature for electrical measurements. The heat transfer module of COMSOL (ver. 4.2) was utilized for 3-D thermal profiling^30^. The material parameters of the EMC were obtained from the data sheet of 832HT (manufacturer: MG Chemicals). The top surface of the EMC was set to a constant temperature of 170 °C, which was experimentally extracted by use of the infrared thermometer after 60 sec exposure of the microwave. In addition, below the bottom of the chip with a thickness of 100 μm, interfacing layer was defined as vaccum^31^_._ The die was defined as a Si wafer and the *κ* of Si was 130 W/m·K.

### TID experiments

After measuring variously sized FinFETs in their initial state, they were exposed to γ-ray irradiation using a ^60^Co source at a constant dose rate of 350 rad/s. The accumulated total radiation dose was 5 Mrad (50 kGy). All terminals of the FinFETs were floated during the irradiation. Then, electrical measurements were carried out within 2 hours to minimize the annealing phenomenon under air ambient at room temperature^3,32^.

## Electronic supplementary material

Supplementary Information

## Data Availability

All the data included in this paper are sharable and available.
