# Supplementary material for: On-Chip Curing by Microwave for Long Term Usage of Electronic Devices in Harsh Environments
Source: Sci Rep. 2018 Oct 8;8:14953. doi: 10.1038/s41598-018-33309-x (PMC6175829; doi:10.1038/s41598-018-33309-x)
Supplement: Supplementary file 1 — Supplementary Information [file 41598_2018_33309_MOESM1_ESM.docx]

**Supplementary Information**

**On-Chip Curing by Microwave for Long Term Usage of Electronic Devices in Harsh Environments**

*Jun-Young Park, Weon-Guk Kim, Hagyoul Bae, Ik Kyeong Jin, Da-Jin Kim, Hwon Im, Il-Woong Tcho, and Yang-Kyu Choi**

School of Electrical Engineering, Korea Advanced Institute of Science and Technology, (KAIST) 291 Daehak-ro, Yuseong-gu, Daejeon 34141, Republic of Korea

* Authors to whom correspondence should be addressed.

Email addresses: [ykchoi@ee.kaist.ac.kr](mailto:ykchoi@ee.kaist.ac.kr)

|  | **Type** | **Cost** | **Recovery speed** | **Chip level recovery** | **Layout efficiency** |
| --- | --- | --- | --- | --- | --- |
| **Ref.**  **3, 5,7** | Heating inside the package | Intermediate | Very fast  (msec) | Impossible | Low |
| **Ref. 9** |  | High | Fast (seconds) |  |  |
| **Ref. 10** |  | Intermediate | NA |  | Intermediate |
| **Ref. 11** |  | High | Fast (seconds) |  | Low |
| **Ref. 12** |  |  | Slow (hours) | Possible | High |
| **Ref. 13** |  |  |  |  |  |
| **Ref. 14** | Heating  outside the package | Low | Slow (hours) |  |  |
| **This work** |  |  | Fast (seconds) |  |  |

**Table S1.** Comparison of the various recovery methods for electronic devices

**Optical image of packaging before and after microwave irradiation**

**Figure S1.** (a-d) Surface image of commercial off-the-shelf chip (model: Z400S, Sandisk), interconnection on PCB, soldering, and PCB, respectively, before the microwave irradiation. (e-h) Surface image the components after the microwave irradiation. There was no any electrical failure such as short or open after the MwC.

**Fabrication process flow of the FinFET on SOI.**

Figure S2. (a) A p-type (100) SOI wafer with a top silicon thickness of 145 nm and a BOX thickness of 400 nm was used as a starting substrate. (b) The thickness of the silicon layer was thinned down from 145 nm to 50 nm. (c) Photolithography process using 0.18 μm technology was employed for the channel (fin) definition of the FinFET. In order to achieve a minimum feature size, photoresist (PR) ashing by oxygen plasma was performed in part. After the PR ashing process, the line width of 180 nm was reduced to 90 nm. (d) After the channel patterning, 5 nm of SiO_2_ was thermally grown, and 100 nm of n^+^ poly-Si was deposited as a gate electrode by use of low-pressure chemical vapor deposition (LPCVD). Then the poly-Si layer was planarized by chemical-mechanical planarization (CMP). (e) Photolithography and the PR ashing process was carried out again for gate definition, and the line width of 180 nm was reduced to 60 nm. (f) After the gate patterning, 100 nm of tetraethyl orthosilicate (TEOS) of 100 nm was deposited by LPCVD, and etched to form 25 nm gate spacers. Then, the source and drain (S/D) were doped with arsenic by ion implataion, the implated dopants were activated by a rapid thermal annealing (RTA) 1000 °C for 5 sec, and the probing pad was opened.

**Grounding method to minimize the damage by static electricity.**

Figure S3. A method to avoid the unwanted damages in the gate insulator caused by the static electricity. (a) Attachment of the G-DPMS film before MwC. An unpackaged die to include FinFETs is fabricated, and a probing metal pad is deposited on a die. (b-c) The G-PDMS film begin to cover the die while the metal pad is connected to ground electrode. (d) The G-PDMS film covers the whole area of the die, and microwave is irradiated for on-chip recovery for 1 min. (e-g) Detachment of the G-PDMS film after MwC. After irradiation of microwave, the G-PDMS film is dettached from the die while the metal probing pad is still connected to ground. (h) The remaining area of the G-PDMS is detached from the die, and the FinFETs on the die are re-measured.
